# Mitigation of Acrylamide in Potato Chips by Pre-drying and Pulsed Electric Field Treatment

**DOI:** 10.3389/fnut.2022.919634

**Published:** 2022-07-11

**Authors:** Caiyun Liu, Rui Zhang, Eugene Vorobiev, Nabil Grimi

**Affiliations:** ^1^School of Health Science and Engineering, University of Shanghai for Science and Technology, Shanghai, China; ^2^ESCOM, TIMR (Transformations Intégrées de la Matière Renouvelable), Centre de Recherche Royallieu, Université de Technologie de Compiègne, Compiègne, France; ^3^National R&D Center for Se-Rich Agricultural Products Processing, Hubei Engineering Research Center for Deep Processing of Green Se-Rich Agricultural Products, School of Modern Industry for Selenium Science and Engineering, Wuhan Polytechnic University, Wuhan, China

**Keywords:** potato chips, pulsed electric fields, vacuum drying, frying, acrylamide

## Abstract

The object of this work was to study the effects of preliminary vacuum drying (VD), pulsed electric field (PEF) treatment, frying temperature on color, oil uptake, and acrylamide (AA) content in fried potato chips. The results of this study indicated that an increase of frying temperature from 120 to 180°C led to a decrease of frying time of around 70% for untreated and PEF pre-treated samples. The color value of L* and a* decreased with the increase of frying temperature, and those values of the sample pre-treated by PEF were significantly higher compared to those obtained from untreated samples. The PEF pre-treatment promoted the reduction of oil content of fried samples by up to 17.6, 14.2, and 16% compared with untreated samples at the frying temperatures of 120, 150, and 180°C, respectively. Higher efficiency was observed by applying the preliminary VD in the case of the frying temperature of 150°C. Furthermore, it was revealed that PEF pre-treatment and preliminary VD application lead to a synergetic effect on the reduction of AA content in potato chips. For example, with the initial moisture ratio of 0.5, pre-dried by VD and pre-treated by PEF, the AA content was noticeably decreased from 2,220 to 311 μg/kg compared to untreated and undehydrated samples at the frying temperature of 150°C. Our findings provide reference for a new pre-treatment to mitigate AA formation and to improve the quality of potato chips.

## Introduction

Acrylamide (AA), a carcinogen formed in heated foodstuffs which was found by the Swedish National Food Administration in 2002, is a significant problem (1). Baked and fried food, such as French fries and bread, is the main source of AA. Several mice experiments on AA carcinogen *in vitro* and *in vivo* systems have been undertaken and they suggested that AA might influence hormonal systems and increase the risk of cancer (2, 3). It has been observed that exposure to AA leads to a DNA damage and the high doses AA would affect the reproduction as well (4). There was a moderate level of AA content in heated protein-rich foods (5–50 μg/kg), but a higher level of AA content was found in carbohydrate-rich foods (150–4,000 μg/kg) (5).

Researchers have recently devoted great attention to understanding the formation of AA and to optimizing the processing conditions to reduce the amount of AA in foodstuffs. One of the major routes for AA formation is the Maillard reaction between amino acid asparagine and reducing sugar (fructose or glucose) at temperatures above 120°C (6–9). Therefore, reducing AA precursors that are free asparagine and reducing sugars could be directed toward for the mitigation of AA. Muttucumaru et al. (10) paid attention to genetic approaches to reducing AA risk, such as changing the cultivated environment to reduce the sugars and/or free asparagine of raw materials. Moreover, vacuolar invertase gene silencing during tuber growth could strongly reduce the sugar and AA content in fried potato strips (11). Baardseth et al. (12) used lactic acid fermentation as a pre-treatment for French fries processing; when the inoculation of lactic acid was done in the blanched potato rods and fermented for between 45 and 120 min, AA content in final product could be reduced by 79 and 94%, respectively. Carrying out the blanching treatment before potato chips processing could significantly limit AA formation (about 64%) by reducing the content of glucose and asparagine, as well as enzyme inactivation in potato slices compared to the unblanched samples (13). Similar results were observed by other researches as well (14). However, the drawbacks of the blanching thermal pre-treatment are high energy consumption and modification of sensorial properties of the final product. During frying process, the oil used for frying can be oxidized and can be converted into acrolein and acrylic acid, which interact with asparagine in the presence of heat and form AA (15). The formation of AA in this way is common in fried foods. To date, the alternative non-thermal technologies were proposed to preserve the product quality. Pulsed electric field (PEF) treatment is well-known for the cell membrane permeabilization that leads to the increase of mass and heat transfer during processing; it has been widely studied in the biomolecular extraction process, drying process, and freezing process (16–21). Pulsed electric field pre-treatment could accelerate the evaporation of moisture and reduce the oil absorption during frying (22). Comparing the impact of PEF pre-treatment and blanching pre-treatment on the AA content of potato crisps, PEF can promote a reduction of AA (≈30%) by removing fructose and asparagine, to a significantly greater degree compared to the AA reduction obtained with blanching (≈17%) (23). Jaeger et al. (24) also stated that PEF treatment could increase the release of reducing sugars and asparagine, which leads to the limiting of Maillard reaction and consequently reduces the AA content. In a recent study, the significant effect of combination of PEF treatment and preliminary vacuum drying (VD) on frying of potato chips was obtained, which elucidates the reduction of frying time, oil uptake, as well as improving the color and texture of the final product (21). However, the combined effects of PEF treatment and VD on AA content of fried sample had never studied before.

The aim of this study was, therefore, to evaluate the effects of preliminary PEF pre-treatment and VD on frying processes of potato disks, paying attention to the preliminary moisture content, PEF pre-treatment, and frying temperature on the formation of AA and other characteristics of potato chips.

## Materials and Methods

### Materials

#### Chemicals

^13^C_3_-acrylamide (98%), AA (99%), formic acid, and methanol were obtained from Sigma–Aldrich (St. Louis, MO, United States); *N*-hexane, acetone, and ethyl acetate were of HPLC grade and obtained from Merck (Darmstadt, Germany). Sodium chloride and diatomite were purchased from Beijing Chemical Factory (Beijing, China); ammonium sulfate was obtained from Tianjing Chemical Factory (Tianjing, China). Ultrapure water (18.2 mΩ⋅cm) was obtained from a Millipore purification system (Billerica, MA, United States).

#### Food Samples

Commercial potatoes (variety EXCELLENCY for mashed and chips) and raw vegetable oil (iSiO4) were purchased in a local supermarket and placed in refrigerator at ≈5°C. All experimental data were collected within 10 days of the purchase. A special cylindrical knife was used to prepare the studied samples with diameter of 25 mm and thickness of 2.5 mm. The initial water content in the potatoes was determined according to the Association of Official Analytical Chemists (AOAC) method (AOAC 2000) by drying about 25 g samples at 105°C in the convection oven (UL50, Memmert, Schwabach, Germany). The initial water content of the sample was 79.71% [wet basis (WB)].

### Treatments

The samples were preliminarily treated by PEF and then dehydrated to *MR*_*V*_ = 0.5 and *MR*_*V*_ = 0.2 by using VD at *P* = 0.3 bar and *T*_*V*_ = 50°C. Lastly the samples were fried at the temperature of 120, 150, and 180°C, and analyzed.

#### Pulsed Electric Fieldtreatment

A PEF generator delivering monopolar pulses (1,500 V–20 A, Service Electronique UTC, France) was used. A sample was placed between the bottom and the upper electrodes in a Teflon cylindrical tube. The PEF treatment was applied using the electric field strength of *E* = 600 V/cm in the series of *N* = 10 trains. Each train consisted of *n* = 100 pulses with pulse duration of *t*_*i*_ = 100 μs, and the time interval between the pulses of Δ*t* = 10 ms. The total PEF pre-treatment time, *t*_*PEF*_ = 0.1 s, applied protocol allowed obtaining the high level of electroporation of potato tissue [e.g., see the data presented in Refs. (25, 26)]. The temperature evolution inside the samples never exceeded 5°C.

#### Preliminary Vacuum Drying

The VD of potato procedure was done in a vacuum oven (Cole–Parmer, 2.3 cu ft, 120 VAC, United States) connected with a vacuum pump (30 kPa, Werie, Rietschle, RTV1, Germany). Untreated (U) and PEF pre-treated samples (PEF) were pre-dried at the VD temperature, *T*_*v*_ = 50°C. Potato chips were removed from the vacuum oven when the moisture ratio were *MR*_*v*_ = 0.5 and *MR*_*v*_ = 0.2 and then used for frying.

#### Frying

The preliminary dried/undried and PEF pre-treated and untreated potato chips were then deep-fried in hot vegetable oil (iSiO4) contained in a beaker at the potato/oil mass ratio of 1/30. The mixture was heated and stirred on a ceramic heating plate C-MAG HS 7 S000 (IKA, France). The temperature of frying *T*_*f*_ = 120, 150, and 180°C were controlled by heating system EST-D6 (IKA, France). The mass of the samples, *m*, during the frying process was periodically controlled. After the frying process, the samples were drained, and blotted using an adsorbent paper.

The moisture ratio, *MR*, of the sample during frying process was calculated as


(1) $$M\,R=\frac{m-m_{\text{d}}}{m_{i}-m_{\text{d}}}$$

where *m*_*i*_ is the mass of initial sample, *m*_*d*_ is the mass of dry matter (DM). It was determined by drying the potato slices at 105°C to a constant weight. The frying process was performed up to the final moisture ratio of *MR*_f_ = 0.1.

### Analysis of the Samples

#### Color

The color of the potato chips was determined by a colorimeter (Konica Minolta CR-321, Japan). The color parameter coordinates L* (whiteness or brightness), a* (redness or greenness), and b* (yellowness or blueness) were used to describe the surface color of samples. The redness parameter, a*, presented the significant variation due to non-enzymatic browning reactions during frying (13).

#### Fat Content

In the presence of frying (*t*_*f*_ ≠ 0), the final oil uptake in the sample, *O*_*f*_, was determined using Soxhlet extraction process with hexane as a solvent according to AOAC Official Methods of Analysis, after the frying process, the sample was put into the convection oven (UL50, Memmert, Germany), dried at 105°C for 24 h. Finally, the oil uptake *O*_*f*_ was calculated as


(2) $$O_{\text{f}}=\frac{m_{\text{o}}}{m_{\text{d}}}$$

where *m*_*o*_ is the mass of oil uptake, *m*_*d*_ is the mass of dry matter (DM).

#### Liquid Chromatography-Tandem Mass Spectrometry Analysis of Acrylamide

Fifty gram of fried sample was taken, pulverized by a food processor (Elfin2.0, Sheng Zheng), and stored frozen at −20°C. Then, 10 μl of 10-mg/L ^13^C_3_-acrylamide internal standard working solution and 10 ml of ultrapure water were added to 2-g of pulverized samples, shaken for 30 mins, and then centrifuged at 4,000 rpm for 10 min (Medifuge™, United States), and the supernatant was collected. For purification, 15 g of ammonium sulfate was added to the sample extraction supernatant, shaken for 10 min to fully dissolve, then centrifuged at 4,000 rpm for 10 min, and the supernatant was collected and used. The collected supernatant was analyzed by LC-MS/MS (at ambient temperature) using the Atlantis C18 columns (5 μm, 2.1 mm × 150 mm ID). The elution was in isocratic mode using a mixture of 0.1% (v/v formic acid and methanol (99.5/0.5, v/v) as mobile phase at a flow rate of 0.2 ml/min for 6.1 min (analytes recorded), injection volume of sample was 25 μl. The electrospray source had the following settings (ESI+): Capillary voltage, 3.5 kV; cone voltage, 40 V; RF lens voltage, 30.8 V; source temperature, 80°C; desolation temperature, 300°C; and ion collision energy, 6 eV. AA was identified by multiple reaction monitoring (MRM). The conditions selected for the MS/MS detection were as follows: Curtain gas 194 (nitrogen): 40 Arb; ion spray, 5,000 V; temperature, 300°C; nebulizer gas (nitrogen), 40 psi; nebulizer gas. The ion m/z55 was used for quantification of AA, and ion m/z58 was used for quantification of ^13^C_3_-acrylamide.

The standard series of working solutions were injected into the LC-MS/MS system, and the peak areas of the corresponding AA and its internal standard were measured. The AA injection concentration (μg/L) of each standard series of working solutions was used as the abscissa. The peak area ratio of AA (m/z 55) and ^13^C_3_-acrylamide internal standard (m/z 58) as the ordinate, draw a standard curve.

The sample solution of 10 μl was injected into the LC-MS/MS system, and the peak area ratio of AA (m/z 55) and ^13^C_3_-acrylamide internal standard (m/z 58) was measured, and the test solution was obtained according to the standard curve. AA content *X* (μg/kg) of the sample (μg/kg) was calculated as


(3) $$X=\frac{B\times f}{M}$$

were *B* is the corresponding mass of AA of the ratio of the peak area of AA (m/z 55) peak and the ^13^C_3_-acrylamide internal standard (m/z 58) peak, ng; *f* is conversion factor of the internal standard addition quantity in the sample (*f* = 1 when the internal standard is 10 μl or *f* = 2 when the internal standard is 20 μl); *M* is sampling quantity when adding the internal standard, g.

### Statistical Analysis

The experiments were replicated at least 5 times. Mean and standard deviation of the data were calculated. The Fisher’s least significant difference (LSD) tests were applied for analysis of the effects of PEF treatment, level of dehydration and frying temperature. For each analysis, the significance level of 5% was assumed. All statistical analyses were performed with a 95% confidence interval. For the derivation of frying rates constant, the software package TableCurve 2D, version 5.01 (Systat Software, San Jose, CA, United States) was used.

## Results and Discussion

**FIGURE 1:**
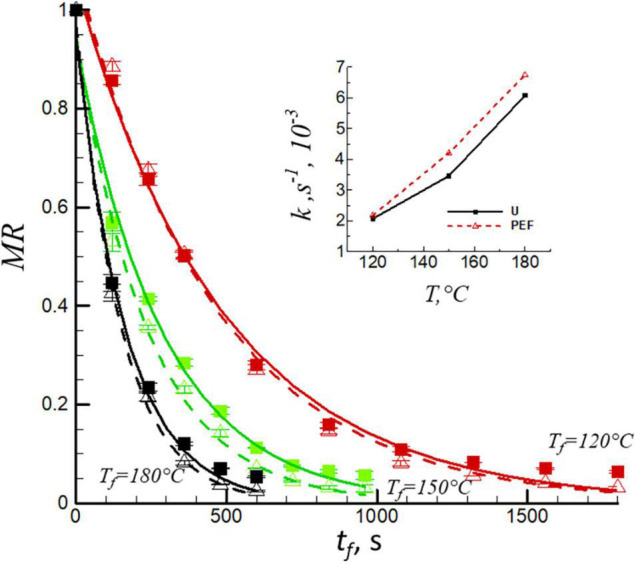
Evaluation of moisture ratio, *MR*, of untreated (solid lines, filled squares) and PEF pre-treated (PEF) (dashed lines, open triangles) samples at different frying temperatures, *T*_*f*_ = 120, 150, and 180°C. The lines were obtained by fitting the data with Eq. 4. Insert of Figure 1 shows the frying rate constant (*k*) for untreated (U) and PEF pre-treated samples.

Figure 1 presents the moisture ratio, *MR*, *vs.* the frying time, *t*, for untreated and PEF pre-treated samples at frying temperatures of 120, 150, and 180°C. An initial rapid decrease in water content was observed for all temperatures, followed by a gradual decrease until a constant moisture value was obtained. As seen from the view of the heat transfer, the increase of drying temperature resulted in a decrease in frying time by nearly 70% for both untreated and PEF pre-treated samples. The higher the frying temperature is, the shorter is the time required for the moisture ratio to reach the equilibrium point. The electroporation obtained by PEF treatment caused a slight decrease of frying time (Figure 1). This finding is in agreement with the previous studies, reporting that PEF processing improves the mass transfer during frying (27, 28). The empirical Henderson and Pabis equation (Eq. 4) was used to fit the curve of moisture ratio as a function of frying time (Figure 1, solid and dashed lines). Software package, TableCurve 2D, version 5.01 (Systat Software, San Jose, CA, United States) was used to obtain the relevant correlation coefficients (*R*^2^) and the frying rates constant, *k*. The *R*^2^ were rather high for untreated and PEF pre-treated samples at all temperatures (*R*^2^ = 0.978–0.901). The values of frying rate constant, *k*, as a function of frying temperature ranged from 2.06 × 10^–3^s^–1^ to 6.07 × 10^–3^s^–1^ and 2.17 × 10^–3^s^–1^ to 6.74 × 10^–3^s^–1^ for untreated and PEF pre-treated samples, respectively (insert to Figure 1). The results demonstrated that the increase in frying temperature and application of PEF pre-treatment caused a significant increase in frying rate constant (*p* < 0.05). Higher frying temperature due to the higher temperature difference between the product and the oil which accelerated the frying rate constant and the moisture evaporation. Similar results were also found in the research of Liu et al. (29). Moreover, PEF pre-treatment caused the electroporation of the sample, which also increased the mass transfer during the frying processes, resulted in the increase of frying rate constant (30).


(4) M⁢R=-A⁢e⁢x⁢p⁢(-k⁢t)

where *A* is the frying coefficient and *k* is the frying rate constant, s^–1^.

Activation energy for diffusion processes is a good indicator of the predominant mechanism. It is the minimum amount of energy that is needed to cause the moisture loss during the frying process (31). In our study, the activation energy of the potato chips during frying process was obtained from Eq. 5, they were 27.94 and 26.518 kJ/mol for untreated and PEF pre-treated samples, respectively. The lower values of *E*_*a*_ for PEF pre-treated sample indicated a less temperature sensitive than the untreated ones, which is in consistent with those reported for drying of the potato cylinders (25.2 ∼ 36.2 kJ/mol) (32). Troncoso and Pedreschi (33) studied the blanch and pre-dried treatment on water loss and oil uptake during the vacuum frying of potato slices, these authors found that the *E*_*a*_ for control, blanched and pre-dried treatment during vacuum frying of potato slices were 24.2, 26.3, and 29.0 kJ/mol, separately. In an another study, *E*_*a*_ = 39.99 and 25.39 kJ/mol were found for fried sweet potato chips with untreated and ultrasound pre-treatments (31).


(5) $$k=k_{0}\,\exp\left(\frac{-E_{a}}{T_{a}\,R}\right)$$

where *k*_0_ is the Arrhenius factor in s^–1^; *E*_*a*_ is the activation energy of moisture diffusion in kJ/mol; *R* represents the universal gas constant (8.314 × 10^–3^ kJ/mol⋅K); and *T*_*a*_ is the absolute temperature of drying air in K.

**FIGURE 2:**
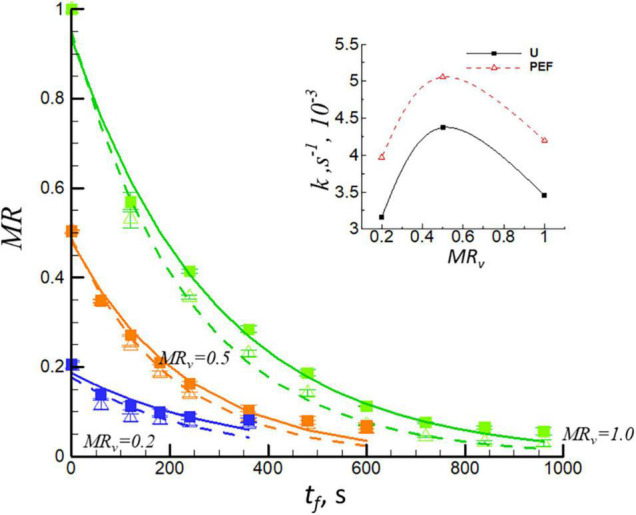
Moisture ratio, *MR*, *vs.* the frying time, *t*_*f*_, for U (solid lines, filled symbols) and PEF pre-treated (dashed lines, open symbols) samples. The samples were preliminary dehydrated by VD to different levels (*MR*_*v*_ = 0.5 and *MR*_*v*_ = 0.2) and then they were fried at 150°C. The curves for undehydrated samples (*MR*_*v*_ = 1.0) are also presented. Insert of Figure 1 shows the frying rate constant (*k*) for untreated (U) and PEF pre-treated samples (PEF).

Figure 2 shows the moisture ratio, *MR*, *vs.* the frying time, *t*_*f*_, for U and PEF pre-treated samples at different levels of *MR*_*v*_ (pre-dried by vacuum drying). The samples were initially dehydrated by VD and then fried at 150°C. The analysis has shown that all frying curves can be satisfactory fitted (with coefficients of determination, *R*^2^ > 0.971) using Eq. 4. The frying time to equilibrium point was decreased with the increase of the level of dehydration (decrease of *MR*_*v*_), which could be explained by the phenomenon of less free water content is available for removal during frying procedure (34). Furthermore, the frying time to obtain *MR*_*f*_ = 0.1 was significantly decreased with PEF pre-treatment. It is evident that there existed a synergetic effect between PEF pre-treatment and preliminary VD. Insert of Figure 2 shows the frying rate constant (*k*) *vs.* the levels of dehydration (*MR*_*v*_) for untreated (U) and PEF pre-treated samples (PEF). It can be seen that the frying rate constant (*k*) increased first (*MR*_*v*_ = 0.5) from 3.45 × 10^–3^s^–1^ to 4.37 × 10^–3^s^–1^ for untreated samples; from 4.19 × 10^–3^s^–1^ to 5.01 × 10^–3^s^–1^ for the PEF pre-treated ones, and then decreased (*MR*_*v*_ = 0.2) from 4.37 × 10^–3^s^–1^ to 3.16 × 10^–3^s^–1^ for untreated samples; from 5.01 × 10^–3^s^–1^ to 3.96 × 10^–3^s^–1^ for the PEF pre-treated ones. The preliminary drying process resulted in a highly heterogeneous structure with a modified moisture distribution; it includes surface diffusion, capillary flow actions, and other mechanisms (35, 36). When dehydrated the moisture ratio of the sample to *MR*_*v*_ = 0.5, it resulted in starch gelatinization, swelling, and softness of sample texture which favors the mass transition during the frying process. Nevertheless, for the sample dehydrated to high level of *MR*_*v*_ = 0.2 before frying (it consists of bound water), it formed a crust on the surface of sample which affected the evaporation of water during frying (37), and thereby resulted in a decrease of frying rate constant compared to *MR*_*v*_ = 0.5 of the sample. Debnath et al. (38) found that the decrease in moisture content before the frying process of the ribbon snack resulted in a lower value of frying rate constant in the same way.

**FIGURE 3:**
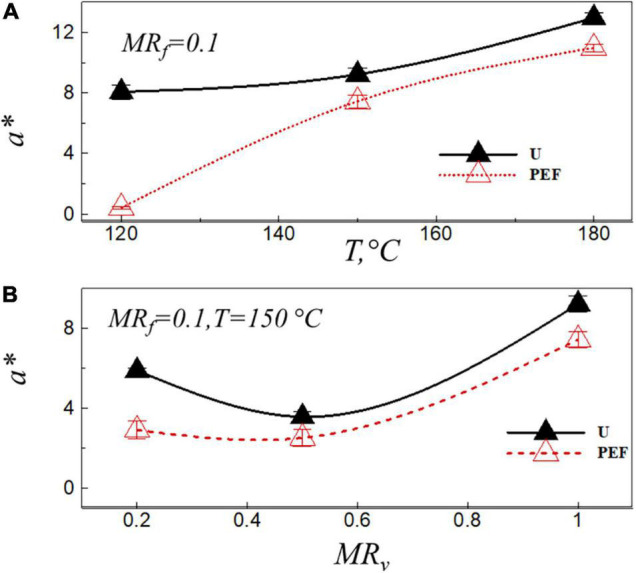
Value of chromatic parameter a* after frying, *MR*_*f*_ = 0.1, *vs.* the frying temperature **(A)**, and the moisture ratio after VD **(B)** for untreated (U) and PEF pre-treated samples (PEF).

Color is related to reducing the sugar content of the potatoes, it is the basic feature of potato chips to appeal to consumer (39). Moreover, according to previous studies, there is a relationship between the AA content and the color coordinate, a* (red-green variation) in the final product (40–43). In addition, several researchers have identified the chromatic parameter, a*, as a useful predictor of AA formation in fried potatoes. They found that the amount of AA content increased with the increase of the chromatic parameter, a*, in general, high values of a* are not desirable (13, 44). Figure 3 shows the value of chromatic parameter, a*, at the final moisture ratio of the fried sample after the frying process, *MR*_*f*_ = 0.1, *vs.* the frying temperature (Figure 3A) and the moisture ratio after VD (Figure 3B) for untreated (U) and PEF pre-treated samples (PEF). As can be seen from Figure 3, the value of a* was significantly (*p* < 0.05) affected by the frying temperature, the preliminary VD and PEF pre-treatment. The coordinate, a*, increased with the increase of the frying temperature, and that of the sample pre-treated by PEF was lower than that of the untreated ones [a* = 8.61 (for untreated samples), a* = 0.41 (for PEF pre-treated samples)] at the temperature of 120°C (Figure 3A). At the frying temperature of 150°C, lowest value of a* was observed at *MR*_*v*_ = 0.5 [a* = 3.57 (U), a* = 2.51 (PEF)] (Figure 3B). The high level of dehydration (*MR*_*v*_ = 0.2) may be resulted in a non-enzymatic browning reaction (Millard reaction) during VD, which increased the value of a* after the frying process (Figure 3B).

**FIGURE 4:**
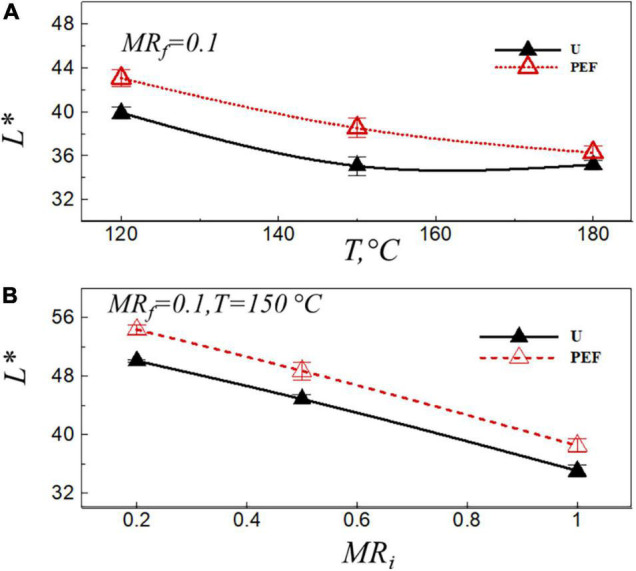
Lightness value, L*, after frying with the final moisture ratio of the sample, *MR*_*f*_ = 0.1, *vs.* the frying temperature **(A)**, and the moisture ratio after VD **(B)** for untreated (U) and PEF pre-treated samples (PEF).

The lightness value, L*, depends on the amount of free water present on the sample surface, which is affected by the preliminary drying, PEF pre-treatment, frying temperature and frying time (40). Figure 4 presents the lightness value, L*, after the frying process at the final moisture ratio of the sample, *MR*_*f*_ = 0.1, *vs.* the frying temperature (Figure 4A), and the moisture ratio after VD (Figure 4B) for untreated (U) and PEF pre-treated samples (PEF). The lightness, L*, decreased with the increase of frying temperature (Figure 4A); this could be associated with the high temperature that increased the level of non-enzymatic browning reactions (Millard reaction) during frying. This result is in coincident with that found in the previous studies (34, 45). PEF pre-treatment produced potato chips with a significant bright color, and this efficiency was more evident with the preliminary VD (Figure 4B). This can be attributed to the shorter frying duration required for the PEF pre-treated samples compared to the untreated ones to obtain the same final moisture ratio of 0.1 (27). Ignat et al. (46) also found that the PEF pre-treatment had a positive effect on the increase of the lightness of samples during the frying process compared to blanched ones.

**FIGURE 5:**
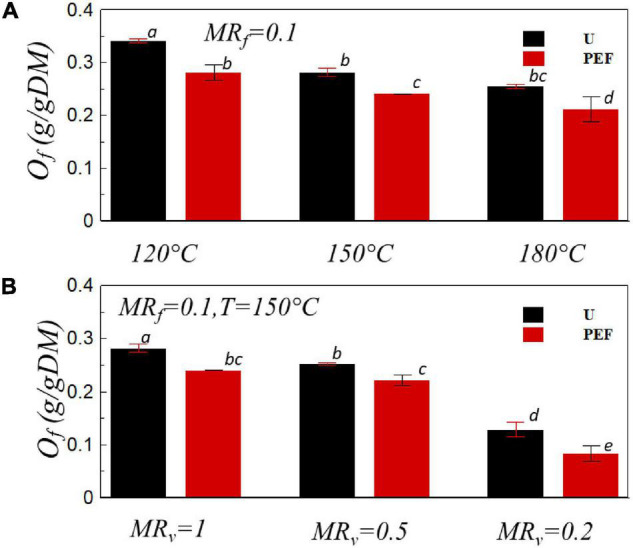
Oil uptake, *O*_*f*_, *vs.* the frying temperature **(A)** and the moisture ratio after VD, *MR*_*v*_ **(B)** for U and PEF pre-treated samples. All samples were fried to *MR*_*f*_ = 0.1 (*p* < 0.05).

The oil content is an important parameter for frying product as it is involved in both oil absorption and water evaporation mechanisms. During the frying process, because of the different temperature between product and frying oil, the moisture would migrate to the surface of the product and evaporate with the concurrent oil that infiltrates into the chips (47, 48). The effects of PEF pre-treatment, temperature, and the combination of PEF and VD on oil contents of final product are presented in Figure 5. For untreated and PEF pre-treated samples, the increase of the temperature decreased the oil content of fried chips as expected (Figure 5A). It can be explained by the increase of the frying rate (Figure 1) with increasing the frying temperature that can keep the water content out of the chips for a longer time, which helps to reduce the final oil content (49). Moreover, the oil uptake of the PEF pre-treated sample was noticeably lower than that of the untreated ones at all temperatures. For example, at the frying temperature of 150°C, the oil uptake for untreated and PEF pre-treated samples were *O*_*f*_ = 0.28 g/gDM and *O*_*f*_ = 0.24 g/gDM, respectively (Figure 5A). It is in accordance with the previously reported results for frying potato products (27, 46). The reduction of oil uptake may be related to the electroporation of potato cell membrane induced by the PEF pre-treatment which enhances the diffusion of water from the core to the surface. At the same time, a thicker water vapor layer on the surface was formed, thereby decreasing the oil absorption during the frying process (50, 51). Furthermore, a smoother surface of the PEF pre-treated samples with less surface area may lead to a reduction in the adhesion of oil during the removal from the surface as it can drip off easier (49).

Figure 5B presents the oil uptake of the sample fried at 150°C with the preliminary VD for untreated (U) and PEF pre-treated (PEF) samples. The oil uptake of the sample significantly decreased with the PEF pre-treatment; furthermore, the reduction of the oil content was more pronounced with the presence of preliminary VD. The oil content of the final product was decreased by up to 71% compared with the sample untreated by PEF and undehydrated by VD. In the previous studies, the drying process effectuated prior to frying led to a significant reduction of oil uptake in different products such as banana chips and French fries (52, 53). A possible reason for the reduction of oil content by preliminary drying during the frying process could be explained by the formation of a crust layer on the surface, which increases the hardness of the sample and prevents the penetration of oil in the product during the frying process (54).

**FIGURE 6:**
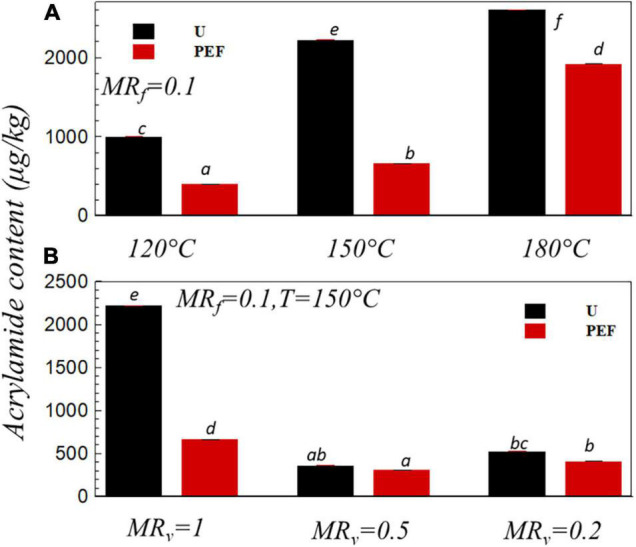
Acrylamide content *vs.* the frying temperature **(A)** and *vs.* the moisture ratio after *VD*, *MR*_*v*_ **(B)** for U and PEF pre-treated samples. All samples were fried to *MR*_*f*_ = 0.1(*p* < 0.05).

Growing evidence show that AA is a process contaminant and neurotoxic of cancer in human (55). AA content in the fried sample (*MR*_*f*_ = 0.1) *vs.* the frying temperature was shown in Figure 6A. It can be seen that the increase of the frying temperature dramatically increased the amount of AA in both untreated and PEF pre-treated samples although the higher oil temperature favored a decrease of the frying time (Figure 1), which, in turn, resulted in a proper environment for the Maillard reaction during the frying process (56, 57). The toxic compound AA was one of the products from the Maillard reaction of reducing sugars and amino acids during thermal processing (58). Hence, the reduction strategies of AA can be explored either by decreasing the AA precursors or by hindering the Maillard reaction pathways (59, 60). The extent of the Maillard reaction depends on the presence of reaction substrate, frying temperature, and frying time (61–65). It was noticed that the higher oil temperature of 170-190°C is adequate to bring the surface temperature above 120°C along with a sufficient moisture loss and thereby favoring the AA formation (66–68). In the study of Liyanage et al. (69), AA content was reduced about 90% with the frying temperature that decreased from 190 to 160°C. Moreover, the application of PEF pre-treatment could significantly decrease the amount of AA by up to 59.7, 70, and 26.15% compared to untreated samples at the frying temperature of 120, 150, and 180°C, respectively (Figure 6A). It can be explained by the increase of mass and heat transfer with the PEF pre-treatment (shorter frying time, Figures 1, 2), as well as the decrease of the Maillard reaction substrate reducing sugar so as to decrease the AA content (70). Genovese et al. (23) compared the effect of the PEF treatment (1.5 kV cm^–1^, 10 ms, 100 Hz) with hot water blanching (85°C, 3.5 min) on AA reduction efficacy in potato chips. They reported AA reduction due to the PEF treatment was nearly 30%, which was significantly higher than the traditional hot water blanching treatment (17%).

Figure 6B presents the AA content in the fried sample (*MR*_*f*_ = 0.1) with the different level of VD dehydration (*MR*_*v*_ = 1.0, *MR*_*v*_ = 0.5, *MR*_*v*_ = 0.2) before frying. First, the amount of AA significantly decreased from 2,220 μg/kg (*MR*_*v*_ = 1.0, U) to 364 μg/kg (*MR*_*v*_ = 0.5, U) and then increased to 524 μg/kg (*MR*_*v*_ = 0.2, U). The same increase/decrease tendency was found in Figure 3B, this phenomenon could suggest that there was a relationship between the AA content and the evolution of the color parameter (carcinogenic a*) of the sample, which is in line with the previous research that reported that the change in color had a good correlation with the AA content in potato chips (13). Mesias et al. (40) analyzed the relativity of samples’ color and AA exposure during the preparation of French fries, they exposed that color parameter (a*) significantly linked with AA, which allows for the assessment of potato-based products’ AA content below or above the benchmark level. In the research of Pedreschi et al. (45), they found that when the sample was pre-dried to moisture content of 60% (wet basis), then fried at the temperature of 180°C, the AA content could be reduced around 22 and 44% compared with the Moms and Frito Lay commercial chips, respectively. When the frying temperature was 150°C and MR_*V*_ = 1.0 (Figure 6B), application of PEF pre-treatment noticeably (*p* < 0.05) decreased the AA content from 2,220 to 666 μg/kg. Nevertheless, there was no significant difference (*p* > 0.05) in AA content in the sample with preliminary dehydrated level of *MR*_*v*_ = 0.5 and *MR*_*v*_ = 0.2 for untreated and PEF pre-treated samples. The pre-treatment with PEF on cell membrane could modify the diffusion of intra- and extra-cellular media during the frying process, and that modification was more complex with the preliminary VD. The combination of VD and PEF pre-treatment before frying modifies the surface structure and forms a low permeability external crust that resists the oil absorption (Figure 5) and reduces the frying time (Figure 2) for achieving same final moisture ratio, which ultimately decreases the heat transfer coefficient resulting in less AA formation (71).

## Conclusion

The effects of preliminary VD and PEF pre-treatment on manufacturing of potato chips were confirmed during this investigation. The frying curves of all temperatures could be properly fitted by the model of Henderson and Pabis (*R*^2^ = 0.978 ∼ 0.901). Cell electroporation phenomenon and structural modifications induced by PEF pre-treatment favors the mass and heat transition during frying processes so as to reduce the frying time and a further improvement on product quality. The application of PEF pre-treatment could also preserve the color of potato chips with a higher L* and a lower a* compared to the untreated samples. Moreover, it was revealed that the oil uptake was distinctly reduced from 0.28 to 0.08g/g DM by the application of PEF pre-treatment (*E* = 600 V, *t*_*PEF*_ = 0.1 s) and preliminary VD (*MR*_*v*_ = 0.2) at the frying temperature of 150°C. The potato chips made of the material pre-treated by PEF and pre-dried by the vacuum processes had noticeably less toxic compound of AA (around decreased 85.9%) than the untreated ones. Thereby, it can be presumed that PEF pre-treatment is able to limit the Maillard reaction, thereby decreasing the AA content. Additional investigations are needed to determine the synergetic mechanism of the combination of pre-drying and PEF pre-treatment on the formation of AA and the absorption of oil in potato chips for industrial applicability.

## Nomenclature

a*: color coordinate redness or greenness at time, *t*; *A*:frying coefficient; b*:color coordinate yellowness or blueness at time, *t; B*: corresponding mass of AA of the ratio of the peak area of the AA (m/z 55) peak and the ^13^C_3_-acrylamide internal standard (m/z 58) peak, ng; *E*: electric field strength, V/cm; *E*_*a*_: activation energy of the moisture diffusion, kJ/mol; *f*: conversion factor of the internal standard addition quantity in the sample; *k*: drying rate constant, s^–1^; *k*_0_: Arrhenius factor, s^–1^; L*: color coordinate whiteness or brightness at time, *t*; *m*: mass of a sample, g; *m*_*i*_: mass of the initial sample, g; *m*_*d*_: mass of the dry matter, g; *m*_*o*_: mass of oil uptake, g; *M*: sampling quantity when adding the internal standard, g; *MR*_*v*_: moisture ratio after VD; *MR*_*f*_: moisture ratio after frying *n*: number of pulses; *N*: number of trains; *O*_*f*_: oil uptake after frying, g/g DM; *R*: universal gas constant, kJ⋅mol^–1^⋅K^–1^; *t*_*f*_: time of frying, s; *t*_*i*_: pulse duration, μs; *t*_*PEF*_: time of PEF treatment, s; Δ*t*: interval between pulses, ms; *T*_*a*_: absolute drying air temperature, K; *T*_*f*_: frying temperature, °C; *T*_*v*_: temperature of preliminary VD; *X*: AA content, μg/kg.

## Data Availability Statement

The original contributions presented in this study are included in the article/supplementary material, further inquiries can be directed to the corresponding authors.

## Author Contributions

CL, EV, and NG contributed to the conception and design of the study. CL organized the database, performed the statistical analysis, and wrote the first draft of the manuscript. All authors contributed to manuscript revision, read, and approved the submitted version.

## Conflict of Interest

The authors declare that the research was conducted in the absence of any commercial or financial relationships that could be construed as a potential conflict of interest.

## Publisher’s Note

All claims expressed in this article are solely those of the authors and do not necessarily represent those of their affiliated organizations, or those of the publisher, the editors and the reviewers. Any product that may be evaluated in this article, or claim that may be made by its manufacturer, is not guaranteed or endorsed by the publisher.

## Abbreviations

AA: acrylamide
PEF: pulsed electric field
MR: moisture ratio
U: untreated
VD: vacuum drying
WB: wet basis (g water/g sample)
LSD: least significant difference
DM: dry matter.
